# A novel nonlinear creep model based on damage characteristics of mudstone strength parameters

**DOI:** 10.1371/journal.pone.0253711

**Published:** 2021-06-24

**Authors:** Bin Hu, Aneng Cui, Kai Cui, Yang Liu, Jing Li

**Affiliations:** 1 School of Resources and Environmental Engineering, Wuhan University of Science and Technology, Wuhan, China; 2 Hubei Key Laboratory for Efficient Utilization and Agglomeration of Metallurgic Mineral Resources, Wuhan, China; China University of Mining and Technology, CHINA

## Abstract

Mudstone interlayer is a weak layer in rock engineering. When it is subjected to continuous stress higher than its damage threshold, due to the dislocation of particles in mudstone crystals and the expansion of cracks, mudstone strength is gradually damaged and deteriorated and the strain gradually increases, thus accelerating the phenomenon of creep damage. In order to describe the characteristics of the whole process of mudstone aging deformation, based on the damage evolution of strength parameters (cohesion and internal friction coefficient) with stress and time in mudstone creep tests, a novel damage nonlinear viscoelastoplastic body (D-NVPB) is proposed through improving traditional plastic element. D-NVPB describes the nonlinear characteristics of the accelerated creep stage of mudstone. With the element combination method, D-NVPB is connected with the Burgers model in series to form a new nonlinear damage creep model (D-NVEP model). The analysis results of creep characteristics theoretically verified the rationality of the model in describing the instantaneous elasticity, viscoelasticity, and nonlinear viscoplastic characteristics of the complete creep curve of mudstone. With the data obtained in the uniaxial compression creep test of mudstone under the action of a stress level of 14 MPa, based on the Levenberg-Marquardt nonlinear least squares method, the fitting calculation was performed through piecewise fitting and overall fitting. The correlation coefficient was 0.9909, which verified the applicability of the model. The obtained model parameters by the identification were used to predict the mudstone creep curve under the stress levels of 13 MPa and 15 MPa. The good prediction results further verified the feasibility of the model. Compared with the traditional creep model, the D-NVEP model can better describe the nonlinear characteristics of the accelerated creep stage and quantitatively display the strength damage evolution process of rock in the creep failure process.

## 1. Introduction

The time-dependent deformation of rock mass due to rheological properties under high in-situ stress conditions is an important factor affecting the long-term stability of rock engineering. Especially, soft rocks shows obvious rheological effects [1, 2]. For example, the mudstone interlayer in the underground coal seam and the limestone mine slope are weak layers because the strength of mudstone is lower than the upper and lower strata and its rheological properties lead to a large deformation [3]. Weak mudstone plays a decisive role in the overall stability of rock mass [4] and is the key object to be considered in the engineering design stage. Mudstone is not an ideal elastic or elastoplastic body. Under the continuous action of a high stress level, original joint fissures (initial damage) in mudstone are gradually compressed and the dislocation within internal crystal grains causes shear slip. The cohesive force and internal friction coefficient gradually decrease, thus weakening the strength of mudstone. With the continuous evolution of the initial damage inside mudstone, the strain gradually increases and finally presents a macroscopic phenomenon of large creep deformation [5].

A complete rock mass creep curve includes three stages: attenuation, constant velocity and accelerated creep [6]. The instability of rock mass involves the accelerated creep stage and the nonlinear viscoplastic deformation caused by accelerated creep stage is one of the hotspots in the studies on rock mass creep. Traditional rheological models cannot however describe the nonlinear characteristics of the accelerated creep stage [7–9]. Relevant models have been extensively explored. Han et al. [10] improved the Burgers model based on the theory of damage mechanics and the improved model well described the nonlinear characteristics of the accelerated creep stage. Xu et al. [11] established a Hohai model which could fully reflect the nonlinear characteristics of accelerated rheology of rocks. Xu et al. [12] conducted a rock creep test with the flexible plate method and provided the basis for engineering design. Tang et al. [13] proposed a nonlinear creep model based on fractional derivative and damage mechanics theory and described the characteristics of rock in different creep stages. Liu et al. [14] introduced damage variables into the Nishihara model to characterize the damage process of the viscosity coefficient, and studied the nonlinear creep characteristics of soft rocks. However, the non-linear characteristics of rock mass creep were seldom explored from the perspective of the deterioration of rock shear strength parameters (cohesion and internal friction coefficient) in the accelerated creep phase (failure phase). In 1985, Yu [15] proposed a crack expansion element which could characterize the unsteady coefficient of friction in the rock mass and opened a new way to describe the nonlinear characteristics of creep. In 2007, based on Yu Qihua’s research, according to Mohr Coulomb’s strength criterion, Yang et al. [16] developed a rheological model considering the damage characteristics of rock mass shear strength parameters and performed the theoretical analysis.

Previous studies on the creep constitutive relationship were mostly based on the principle of damage mechanics from the perspectives of the elastic modulus, viscosity coefficient of rock mass and separate damage factors. In the study, based on previous studies [17–19], through mudstone creep tests, from the perspective of the deterioration of rock mechanical parameters with stress and time, the damage characteristics of two mudstone parameters (cohesion and internal friction coefficient) in the accelerated creep stage are explored in order to quantitatively reveal the rock strength damage evolution in the failure process. A novel nonlinear creep model is established to study the time-dependent characteristics of rock deformation.

## 2. Comparative analysis of full stress-strain curve and creep curve of uniaxial compression tests

Defects of different scales such as pores, joints, cracks and dislocations are naturally formed in rocks in the long geological history process and uneven initial damages exist in rocks [20, 21], but the development of initial damage has a stress threshold. Under the continuous action of the stress level above this threshold, the uneven and localized damage evolution phenomenon occurs, thus resulting in aging deformation and failure [22].

**Fig 1:**
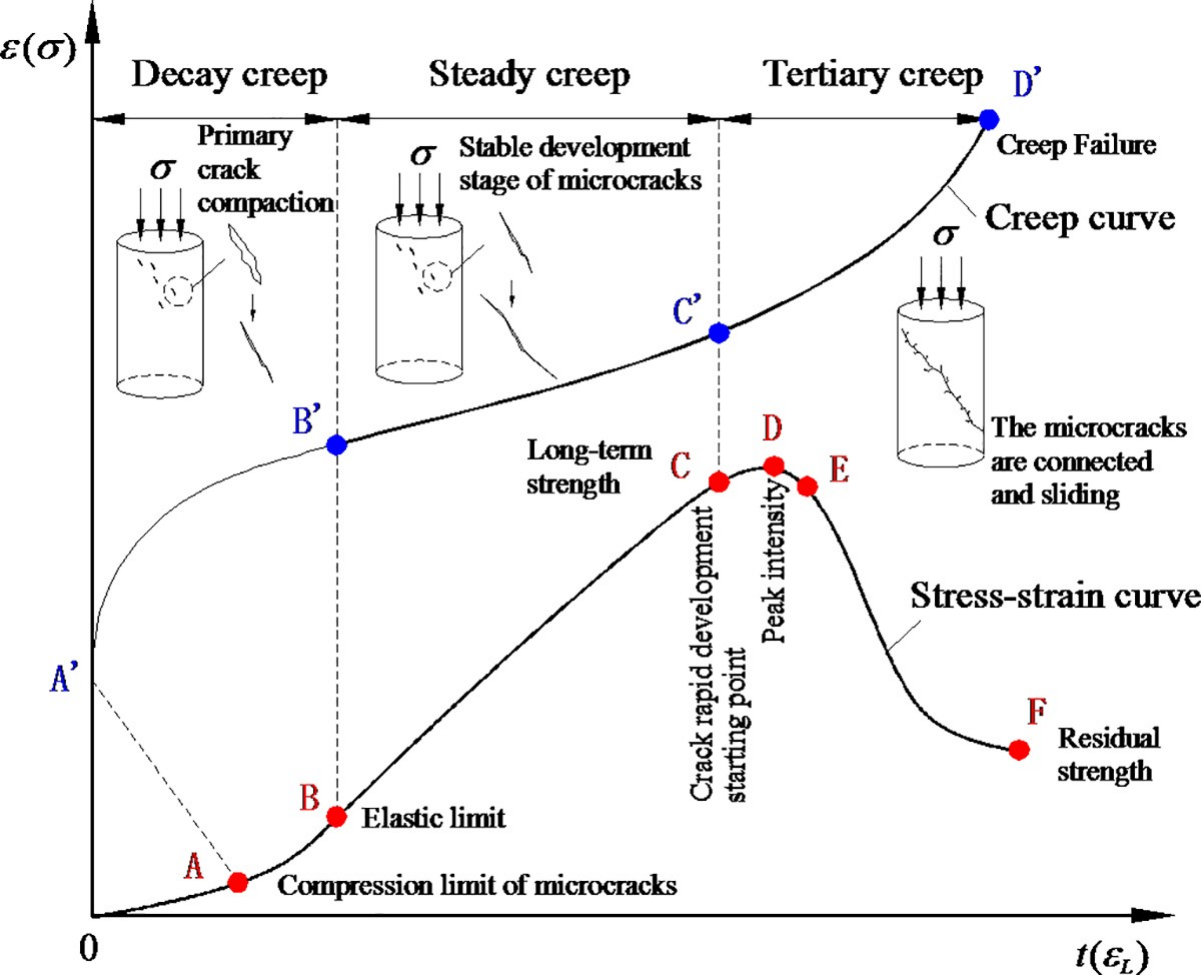
Comparison of total stress-strain curve and creep curve.

The comparative analysis results of the full stress-strain curve and the creep curve shown in Fig 1 are summarized below. Firstly, the OA section corresponds to the OA’ section. When the stress level is low, at the moment of loading, partial originally opened structural surfaces and micro-cracks in rock specimens are closed, thus resulting in instantaneous elastic deformation. Secondly, AB corresponds to the A’B’ section. Under the continuous action of stress, pores and cracks in rocks are gradually compacted and the deformation increases, but the strain rate gradually decreases and enters the attenuation creep stage. Thirdly, when the stress level increases and exceeds the elastic limit (Point B) of rocks, the deformation develops from elastic and viscoelastic stages to the micro-elastic crack stage. Under the continuous action of stress, cracks expand stably and rocks are stable. Fourthly, Point C indicates the long-term strength value of the rock and was also called the “third yield point” of rocks by Chen [23]. Some scholars [24–26] believed that the damage threshold of the rock was its long-term strength value. When the stress is higher than the long-term strength value, the full stress-strain curve passes through Point C and slippage and dislocation occur between joints. Under the continuous action of stress, cracks rapidly expand, cross each other, form main cracks along the direction of the principal stress, and result in continuous and irreversible viscoplastic strain and even shear failure [27].

## 3. Damage characteristics of rock cohesion and internal friction coefficient

According to the Mohr-Coulomb criterion, the shear strength of a rock is determined by the cohesion and internal friction coefficient. When the stress level is higher than the damage threshold of a rock, crystal grains and cracks inside rocks are dislocated and slide, thus decreasing the cohesion and internal friction coefficient of rocks.

Based on previous creep test results [28], the specimen was a gray-black mudstone interlayer in the Qinyuan Coal Mine in Baoji City and composed of clay minerals and scaly clastic minerals. In the tests of three groups, creep time was respectively 100 h, 400 h, and 1000 h. In each group, the load was applied according to 50%~85% of the uniaxial compressive strength value and the loading stress gradient was 5%. The XTR-01 type computer was used to control the electro-hydraulic servo testing machine and the time-varying cohesion and internal friction angle were measured with the single specimen method.

The accelerated creep characteristics of mudstone were analyzed as follows. Firstly, the long-term strength value of the mudstone was experimentally determined to be 9.44 MPa. Secondly, under the action of a stress level of 10 to 11 MPa, the creep curve passed through the initial elastic strain and attenuated creep stages and steady-state creep occurred in a constant creep rate. The higher the stress level was, the faster the steady-state creep rate was. The creep test did not enter the accelerated creep stage after 1000 h. Thirdly, accelerated creep occurred when the stress level was 12~17 MPa. In the stage, the strain gradually increased. When the over-stress threshold difference *σ*-*σ*_s_ increased, the total creep time history became shorter and the specimen broke faster. The viscoplastic strain continuously increased in the accelerated creep stage and was the macroscopic performance of the strength damage of the specimen, suggesting the degradation process of rock mechanical properties. When the stress level was higher than the damage threshold of mudstone, the strength parameter of mudstone was related to the duration of high stress and the over-stress threshold difference. After sorting out the test data, the relationships between cohesion, internal friction coefficient, overstress threshold difference and time are obtained (Table 1).

**Table 1:** Relationships between cohesion, internal friction coefficient and (*σ*-*σ*_s_)*t*.

(*σ*-*σ*_s_)*t*/MPa·h	*c*/MPa	*tan*φ
0	4.64	0.808
100	4.27	0.795
200	4.01	0.783
300	3.90	0.774
400	3.76	0.765
500	3.67	0.751
600	3.57	0.739
800	3.48	0.722
1000	3.27	0.709
1200	3.25	0.697
2000	2.72	0.682

According to the above analysis results of creep damage mechanism, in the creep test, as new damage occurred inside the rock, the cohesive force and internal friction coefficient decreased with the increase in the overstress threshold difference and the creep time. The immediate shear strength parameters (cohesion and internal friction coefficient), therefore, meet the following equations [29]:

$$c(\sigma -\sigma_{S},t)=(C_{0}-C_{S})(1-D_{c})+C_{S},$$ (1)

$$f(\sigma -\sigma_{S},t)=(f_{0}-f_{S})(1-D_{f})+f_{S},$$ (2)

where *C*_0_ and *f*_0_ are respectively the initial instantaneous strength of cohesion and internal friction coefficient; *C*_s_ and *f*_s_ are respectively the long-term strength values of cohesion and internal friction coefficient; *D*_c_ and *D*_f_ are respectively the damage variables of cohesive force and internal friction coefficient. It is assumed that *D*_c_ and *D*_f_ respectively meet the exponential function relationship between the overstress threshold difference and time:

$$D_{c}=1-\exp[-(\sigma -\sigma_{s})t/X],$$ (3)

$$D_{f}=1-\exp[-(\sigma -\sigma_{s})t/Y],$$ (4)

where *X* and *Y* are respectively the attenuation coefficients of cohesion and internal friction coefficient and related to lithology. It can be seen from Eqs (3) and (4) that under the continuous action of stress, the larger attenuation coefficients (*X* and *Y*) correspond to the smaller damage variables and the slower attenuation. The smaller values of *X* and *Y* correspond to the larger damage variables and faster attenuation. Different values of *X* and *Y* can reflect the difference in the sensitivity of cohesion and internal friction coefficient to time and stress.

In the creep test, each level of stress was designed based on the uniaxial compressive strength. The variation of the over-stress threshold difference is therefore small and the damage variables are mainly affected by creep time *t*. When *t* = 0, the damage variable is 0; When *t* approaches infinity, damage variables approaches 1.

Substituting Eqs (3) and (4) respectively into Eqs (1) and (2) gives:

$$c(\sigma -\sigma_{S},t)=(C_{0}-C_{S})\exp[-(\sigma -\sigma_{S})t/X]+C_{S},$$ (5)

$$f(\sigma -\sigma_{S},t)=(f_{0}-f_{S})\exp[-(\sigma -\sigma_{S})t/Y]+f_{S}.$$ (6)

**Fig 2:**
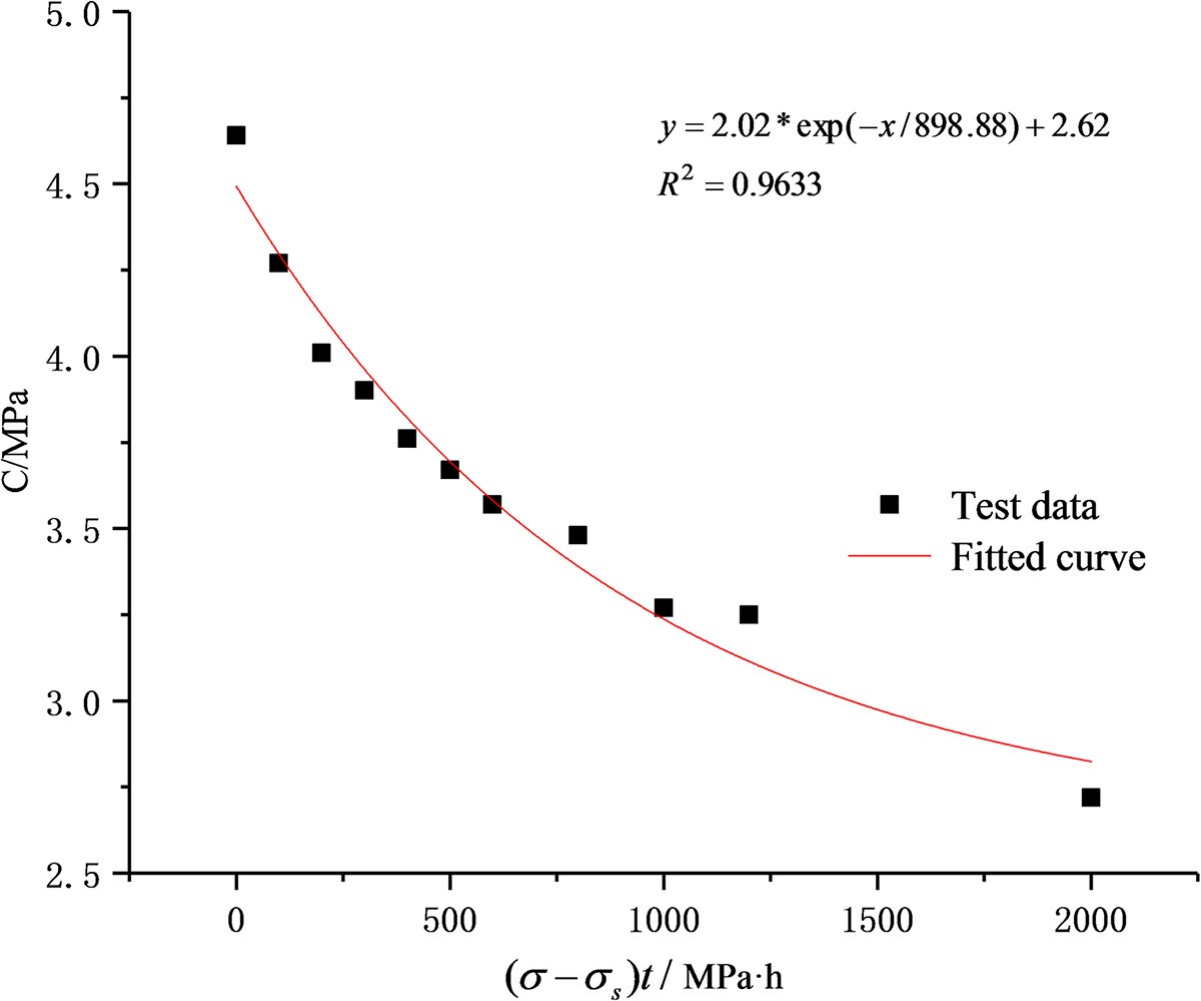
Relationship between cohesion and (*σ*-*σ*_s_)*t*.

**Fig 3:**
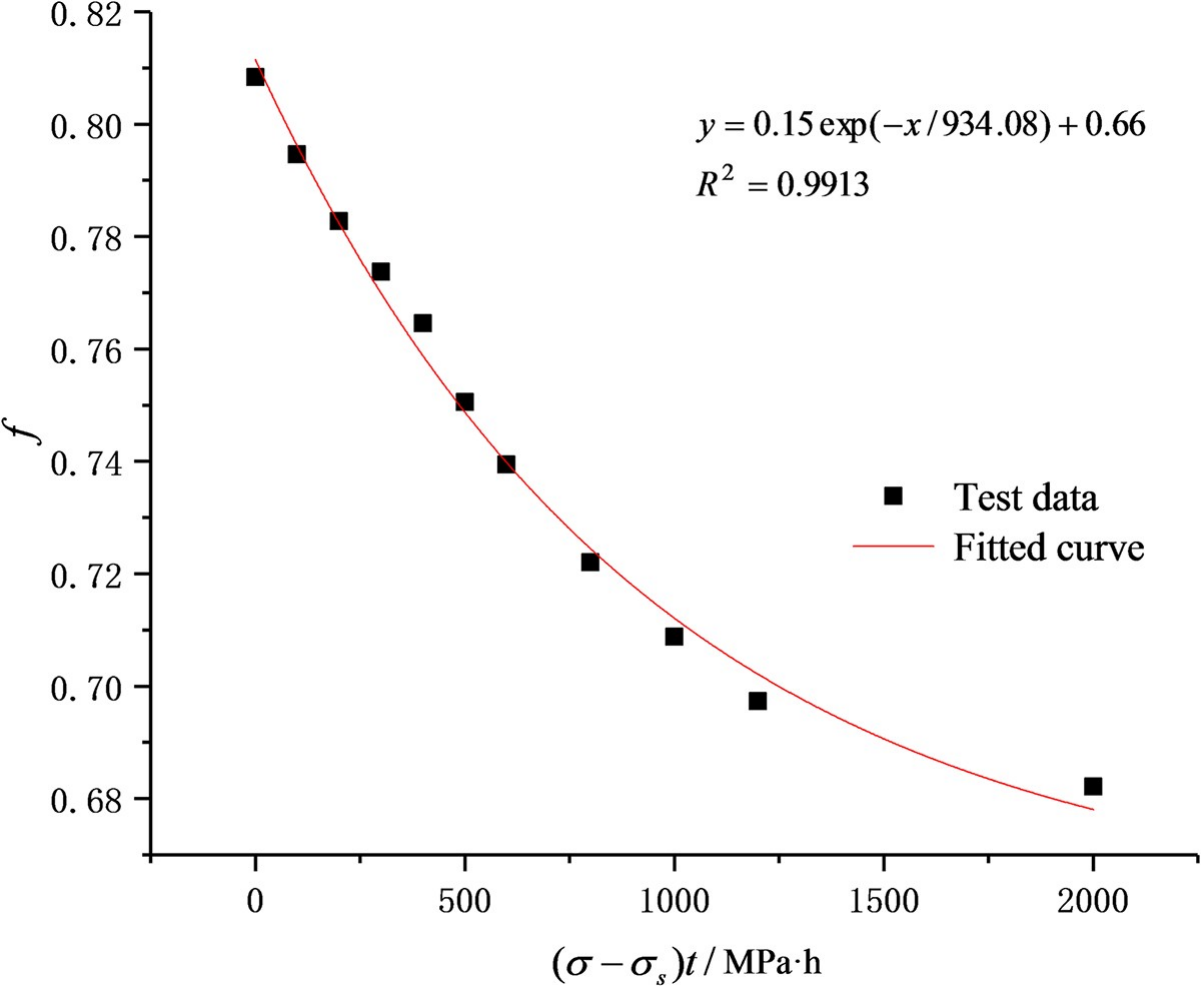
Relationship between internal friction coefficient and (*σ*-*σ*_s_)*t*.

According to the test results, *C*_0_ and *C*_s_ are respectively 4.64 MPa and 2.62 MPa and *f*_0_ and *f*_s_ are 0.81 and 0.66, respectively. Eqs (5) and (6) are used to fit the test data in Table 1 and the results are shown in Figs 2 and 3. *X* and *Y* are respectively 898.88 and 934.08 and the correlation coefficients are larger than 0.95, indicating the better fitting results. In other words, the damage trends of cohesion and internal friction coefficient meet the exponential function relationship.

## 4. Establishing a nonlinear rheological model considering the damage of rock strength parameters

### 4.1. Steady-state creep stage

**Fig 4:**
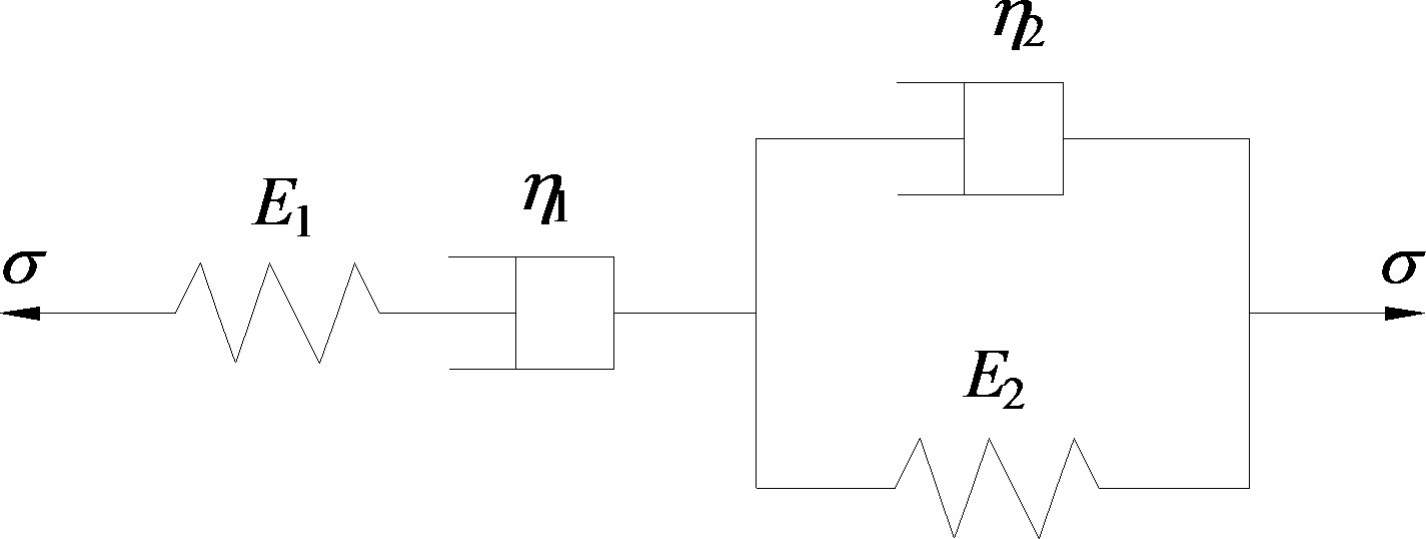
Schematic diagram of Burgers creep model.

As indicated in the above analysis, when the stress level is lower than the long-term strength of rocks, only steady-state creep deformation occurs at the beginning of the first and second creep stages. A classic Burgers body is composed of Maxwell body and Kelvin body connected in parallel. This model can well describe the characteristics of rock rheology, including initial instantaneous elastic deformation, deceleration creep and constant velocity creep [29]. The mechanical model is shown in Fig 4.

The constitutive equation of Maxwell body and Kelvin body and the strain relationship for the models connected in series are expressed as:

$$\begin{array}{l} \varepsilon_{1}=\frac{\sigma_{0}}{E_{1}}[1-\exp(-\frac{E_{1}}{\eta_{1}}t)]\\ \varepsilon_{2}=\frac{\sigma_{0}}{E_{2}}+\frac{\sigma_{0}}{\eta_{2}}t\\ \varepsilon =\varepsilon_{1}+\varepsilon_{2} \end{array}\}.$$ (7)

Based on the above equations, under the action of constant stress σ0, the creep equation of Burgers body is expressed as:

$$\varepsilon =\frac{\sigma_{0}}{E_{2}}+\frac{\sigma_{0}}{\eta_{2}}t+\frac{\sigma_{0}}{E_{1}}[1-\exp(-\frac{E_{1}}{\eta_{1}}t)].$$ (8)

### 4.2. Nonlinear viscoelastoplastic body (D-NVP body) considering the damage of rock strength parameters

**Fig 5:**
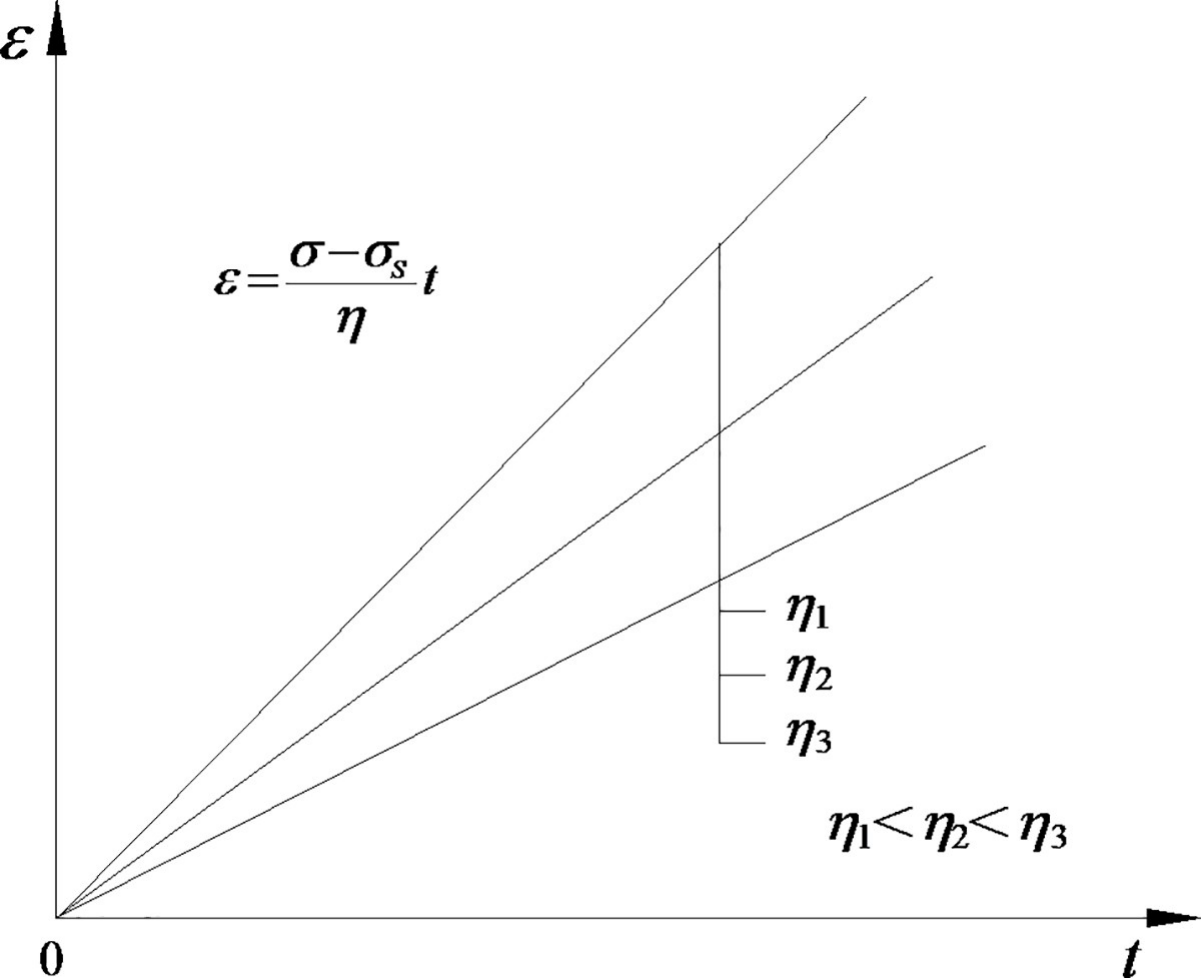
N|Y strain time curve.

**Fig 6:**
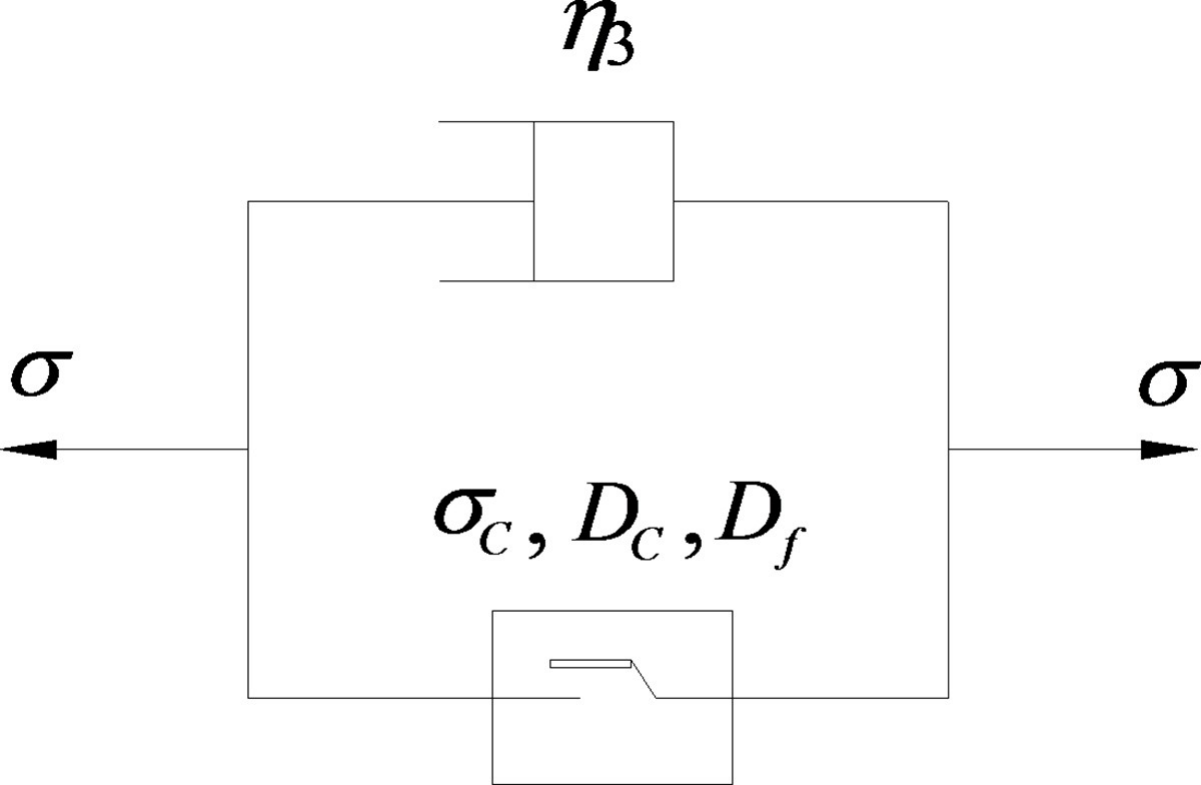
D-NVP volume schematic.

The traditional viscoplastic body (N|Y body) is composed of a Newtonian viscous pot and a friction plate connected in parallel. As shown in Fig 5, its strain has a linear relationship with time, which is inconsistent with the nonlinear characteristics of the accelerated creep stage. As indicated in the above experimental analysis, according to the obtained damage trends of rock cohesive force and internal friction coefficient with creep time, through improving the friction plate in the N|Y body, the viscoplastic body considering the damage of rock strength parameters is established. When the stress level is higher than the long-term strength of rocks, the nonlinear characteristics of viscoplastic deformation (accelerated creep stage) are formed due to the decrease in rock strength. The creep model is shown in Fig 6.

Based on the Mohr-Coulomb criterion of rock failure, it is assumed that the shear friction strength of the rock meets the following equation:

$$\sigma_{M}=\sigma_{S}+h(C_{0}-C_{S})(1-D_{c})+g(f_{0}-f_{S})(1-D_{f}),$$ (9)

where *h* and *g* are the characteristic parameters of the model and related to lithology.

Eq (9) can be simplified as:

$$\sigma_{M}=\sigma_{S}+h(1-D_{c})+g(1-D_{f}).$$ (10)

In Eq (10), when *t* = 0, *σ*_*M*_ = *σ*_0_; when *t*→∞, *σ*_*M*_ = *σ*_*s*_.

According to the parallel connection of the components, the creep differential equation of the D-NVP body is expressed as:

$$\sigma =\sigma_{S}+h(1-D_{c})+g(1-D_{f})+\eta \dot{\varepsilon }.$$ (11)

In Eq (11), when *g* is 0, the D-NVP body degenerates into a nonlinear viscoplastic body considering only the damage of the internal friction coefficient, which is consistent with the previous modeling idea [12]; when *g* and *h* are both equal to 0, it degenerates into traditional parallel combination of plastic elements and viscous elements.

Based on Eqs (3) and (4), by integrating Eq (11), the creep equation of D-NVP body is obtained as:

$$\varepsilon =\{\begin{array}{l} 0,\sigma <\sigma_{S}\\ \frac{\sigma -\sigma_{S}}{\eta_{3}}t+\frac{Xh}{\eta_{3}(\sigma -\sigma_{S})}\exp[-\frac{\left(\sigma -\sigma_{S}\right)}{X}t]+\\ \frac{Yh}{\eta_{3}(\sigma -\sigma_{S})}\exp[-\frac{\left(\sigma -\sigma_{S}\right)}{Y}t],\sigma \geq \sigma_{S} \end{array}.$$ (12)

### 4.3. Analysis of creep characteristics of D-NVP body

According to Eq (11), the creep rate equation of D-NVP body is obtained as:

$$\dot{\varepsilon }=\frac{1}{\eta }\{\sigma_{0}-[\sigma_{S}+h(1-D_{c})+g(1-D_{f})]\}.$$ (13)

Since Eq (10) is a decreasing function of time, Eq (13) is greater than 0, indicating that the creep rate is greater than 0 and that the strain increases with time.

According to Eq (13), the creep acceleration equation is obtained as:

$$\ddot{\varepsilon }=\frac{1}{\eta }[hD_{c}^{'}(t)+gD_{f}^{'}(t)].$$ (14)

**Fig 7:**
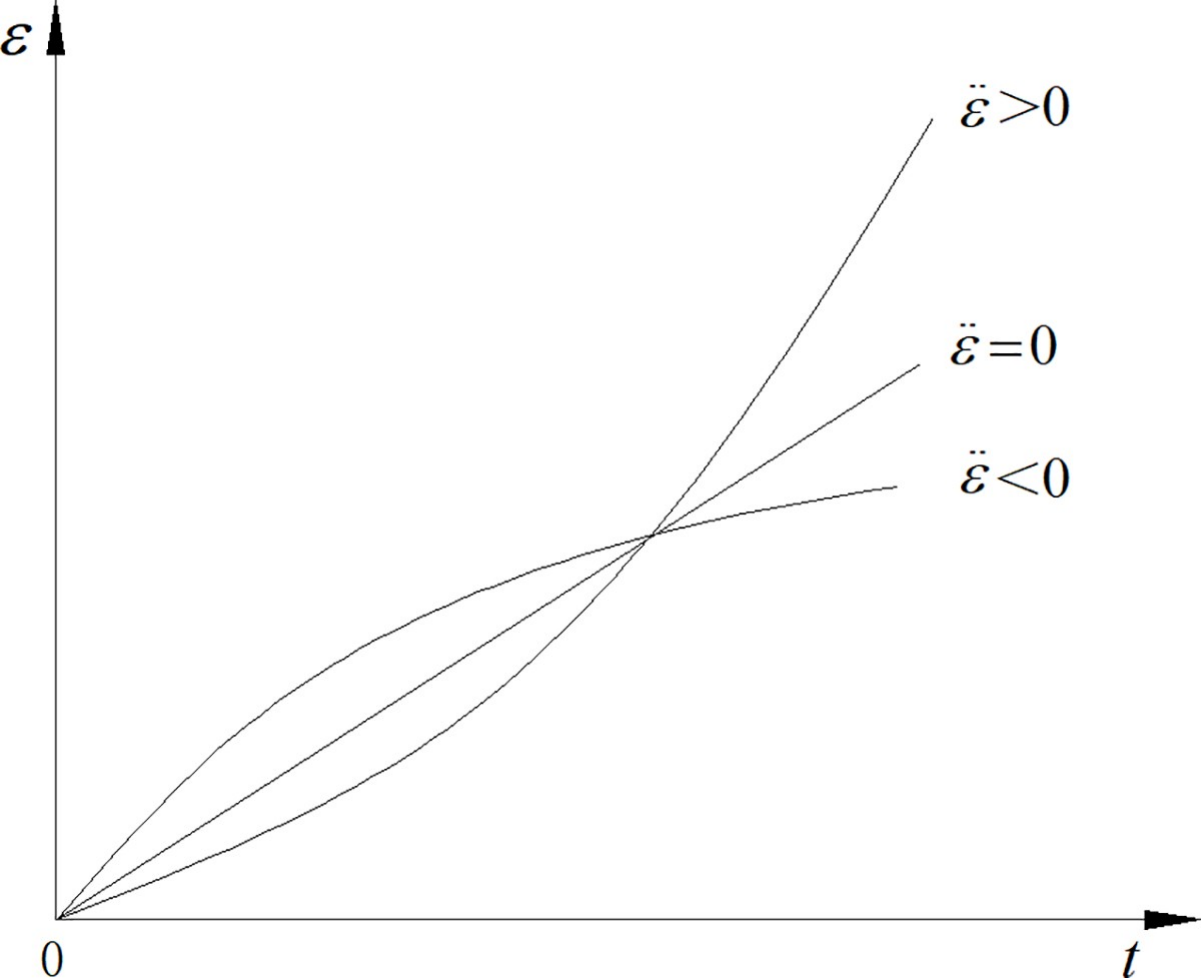
Creep characteristics of D-NVP.

Since the damage variable is an increasing function of time, its first derivative is a positive value. When the characteristic parameters of rock (*h* and *g*) are greater than 0, the creep acceleration is a positive value and the strain increases with time. The larger values of *h* and *g* correspond to the greater creep acceleration, the shorter accelerated creep stage, and the more significant plasticity of the creep failure. The smaller values of *h* and *g* correspond to the longer accelerated creep stage and the more significant rheological characteristics (viscoplasticity) of the creep failure. Under different values of *h* and *g*, the creep acceleration presents three possibilities, as shown in Fig 7. Through the above analysis of the stability of the nonlinear damage viscoplastic model, its description of the nonlinear characteristics of the accelerated creep stage is theoretically verified.

### 4.4. Non-linear viscoelestoplastic creep model based on the damage characteristics of mudstone strength parameters (D-NVEP creep model)

**Fig 8:**
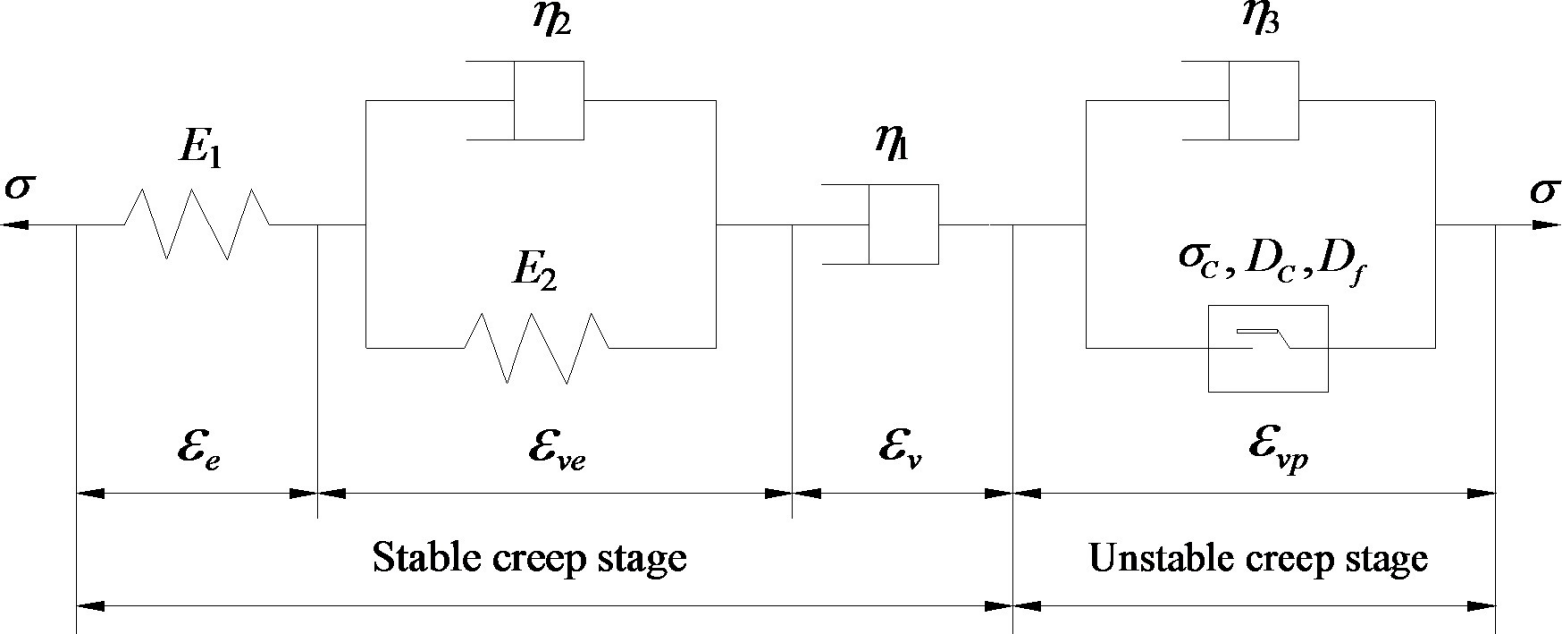
Schematic diagram of D-NVEP model.

The component combination method [30] is used to establish the D-NVEP creep model. First, the differential equation of the nonlinear part is established. Then, the creep equation of this part is obtained through integration. Finally, the obtained creep equation is superimposed with the creep equation of the classical component model. The one-dimensional creep model is shown in Fig 8.

The creep equation under one-dimensional stress state is:

$$\varepsilon =\{\begin{array}{l} \frac{\sigma_{0}}{E_{2}}+\frac{\sigma_{0}}{\eta_{2}}t+\frac{\sigma_{0}}{E_{1}}[1-\exp(-\frac{E_{1}}{\eta_{1}}t)],\sigma <\sigma_{S}\\ \frac{\sigma_{0}}{E_{2}}+\frac{\sigma_{0}}{\eta_{2}}t+\frac{\sigma_{0}}{E_{1}}[1-\exp(-\frac{E_{1}}{\eta_{1}}t)]+\frac{\sigma_{0}-\sigma_{S}}{\eta_{3}}t+\\ \frac{Xh}{\eta_{3}(\sigma -\sigma_{S})}\exp[-\frac{\left(\sigma -\sigma_{S}\right)}{X}t]+\\ \frac{Yg}{\eta_{3}(\sigma -\sigma_{S})}\exp[-\frac{\left(\sigma -\sigma_{S}\right)}{Y}t],\sigma \geq \sigma_{S} \end{array}.$$ (15)

## 5. Parameter identification and model verification

### 5.1. Levenberg-Marquardt nonlinear least square method (L-M method)

The identification methods of rheological model parameters mainly include analytical methods and numerical methods. The LM method is a nonlinear optimization algorithm that combines the Newton method with the gradient descent method. Compared with the experimental design method, regression analysis method, least square method, optimization identification method and intelligent identification method, the LM method reduces the probability of the local minimum of the objective function and has the fast convergence advantage of the Newton method. By setting the damping factor, the non-convergence phenomenon in the fitting due to the singular coefficient matrix in the traditional least squares method is avoided. When the damping factor is 0, the LM method becomes the Gauss-Newton method. When the damping factor is large, it becomes the optimal step calculation of the gradient descent method and has a global search function.

The L-M method uses the nonlinear function *y* = *f*(*x*, *d*) to transform the solution process into the iterative calculation process of n sets of least squares, and the residual square sum *e* is calculated with *n*(*x*_i_, *y*_i_):

$$e=\sum_{i=1}^{p}{\left[Y_{i}-f(x_{i},d)\right]}^{2},$$ (16)

where *x* is a vector of independent variables and *d* is a vector of unknown parameters.

The iterative calculation result obtained under the minimum residual sum of squares is regarded as the approximate analytical solution of the objective function. In a small neighborhood calculated in the m-th iteration, assuming that *f*_m_(*x*, *d*^(m)^) is approximately a linear function, then,

$$f(d^{\left(m\right)}+\delta^{\left(m\right)})\approx f(x_{i},d^{\left(m\right)}+\delta^{\left(m\right)})+A^{\left(m\right)}\delta^{\left(m\right)},$$ (17)

where

$$A^{\left(m\right)}={\left[\frac{\partial f(x,d)}{\partial d_{j}}\right]}_{d-d^{\left(m\right)}},j=1,2,\ldots n.$$ (18)

Then *m*+1 iterations give:

$$d^{\left(m+1\right)}=d^{\left(m\right)}+\delta^{\left(m\right)}.$$ (19)

If

$$\|y-f(x,d^{\left(m+1\right)})\|=\underset{\delta^{k}}{\min}d^{\left(m\right)}\|A^{\left(m\right)}\delta^{\left(m\right)}-e^{\left(m\right)}\|,$$ (20)

according to

$$A^{\left(m\right)}\delta^{\left(m\right)}=e^{\left(m\right)},$$ (21)

the least squares solution is obtained as:

$$\delta^{\left(m\right)}{}_{LM}={\left[{\left(A^{\left(m\right)}\right)}^{'}A^{\left(m\right)}+\lambda^{\left(m\right)}I\right]}^{-1}{\left(A^{\left(m\right)}\right)}^{'}e^{\left(m\right)},$$ (22)

where *λ*^(m)^I is the damping term and *λ*^(m)^ is the damping factor.

### 5.2. Model verification

When the stress level is lower than the long-term strength of rocks, the D-NVEP creep model degenerates into the classic Burgers model, which can better describe the attenuation creep and steady-state creep characteristics of soft rocks. In order to verify the applicability of the D-NVEP creep model in describing the nonlinear characteristics of the accelerated creep stage, with the uniaxial creep test data of mudstone under the stress level of 14 MPa in the previous study [16], the fitting calculation is performed through piecewise fitting and overall fitting. Based on n groups of test values (*t*_i_, *ε*_i_), *E*_1_, *E*_2_, *η*_1_, *η*_2_, *η*_3_, *h*, and *g* are selected as optimization parameters and related parameters are identified with LM-nonlinear least squares method in Origin software.

**Fig 9:**
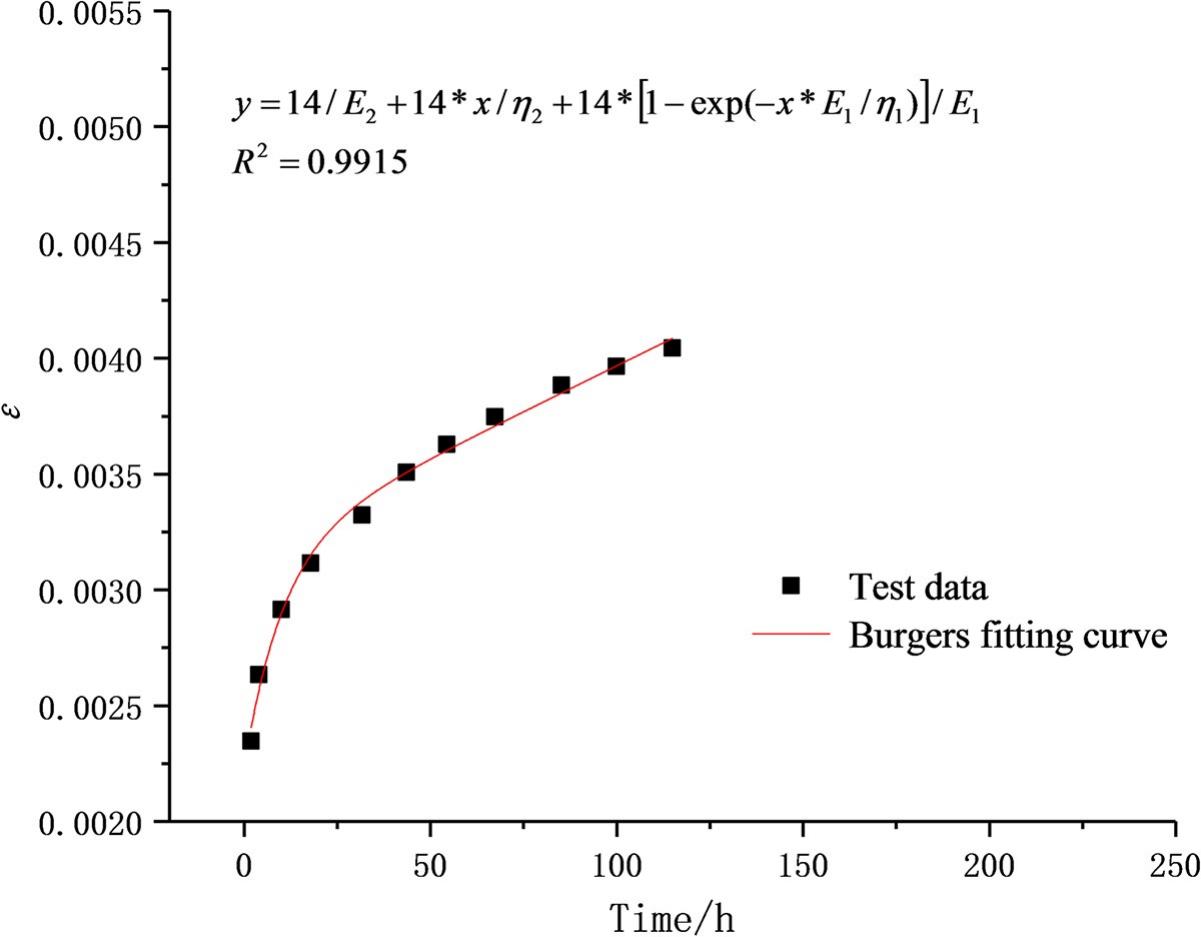
Fitting curve in stable creep stage.

**Fig 10:**
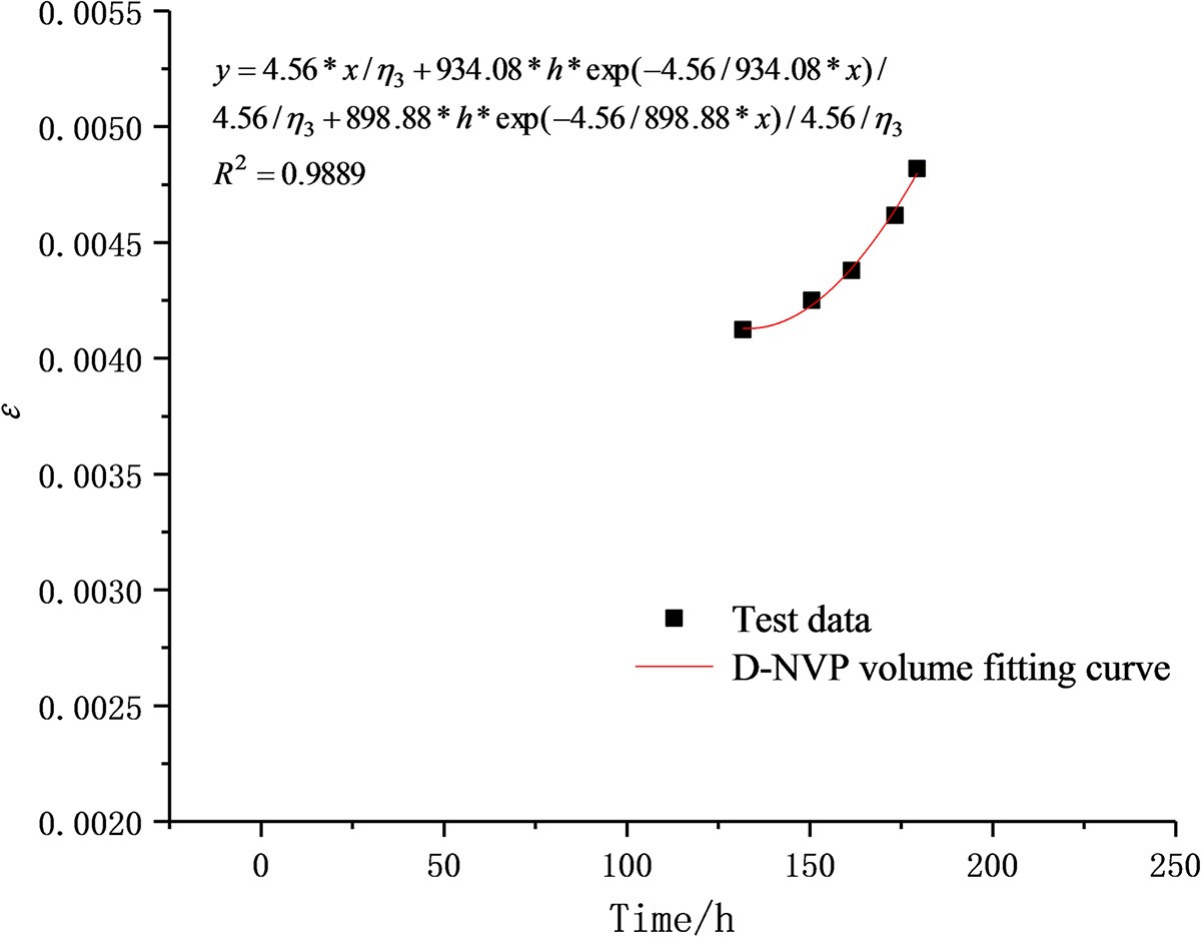
Fitting curve in unsteady creep stage.

**Fig 11:**
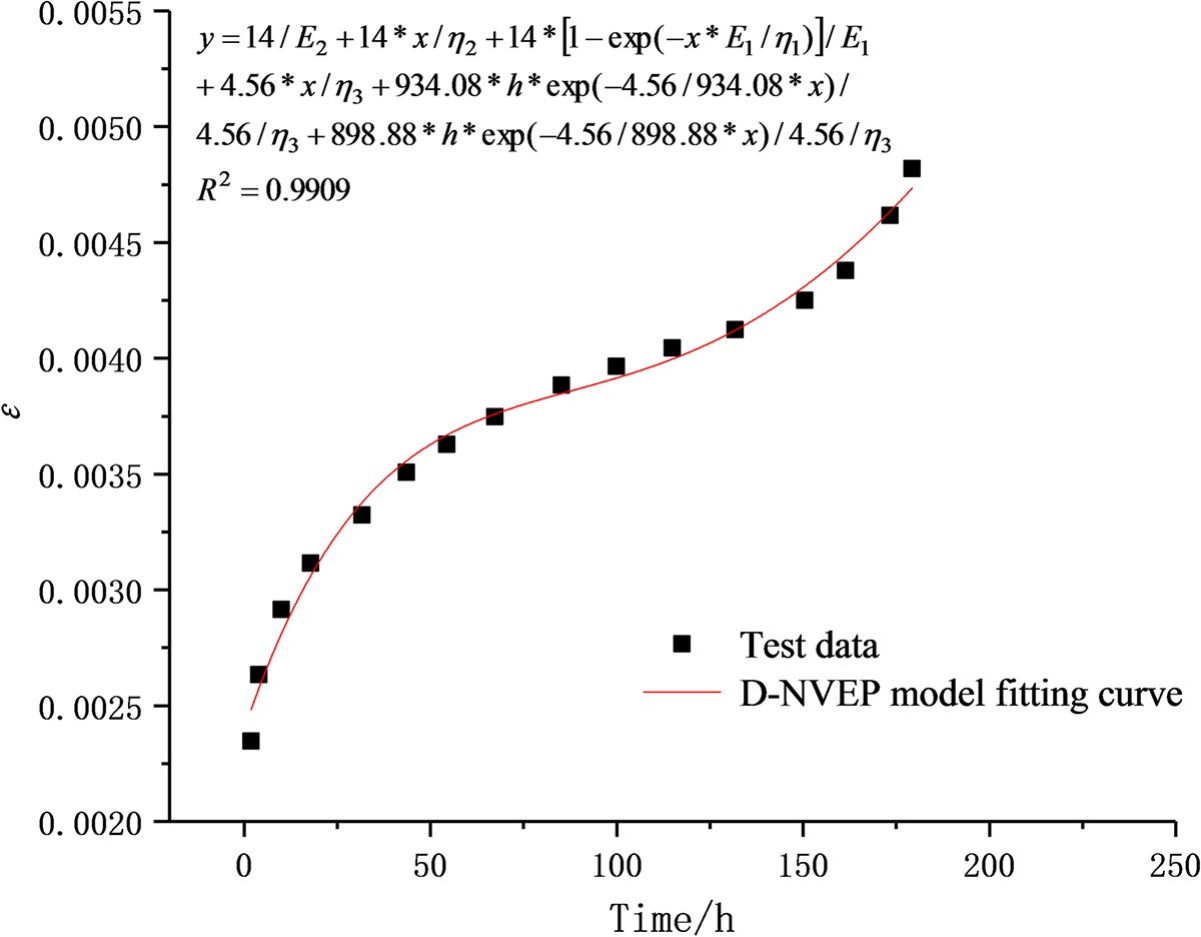
Fitting curve of D-NVEP model.

**Fig 12:**
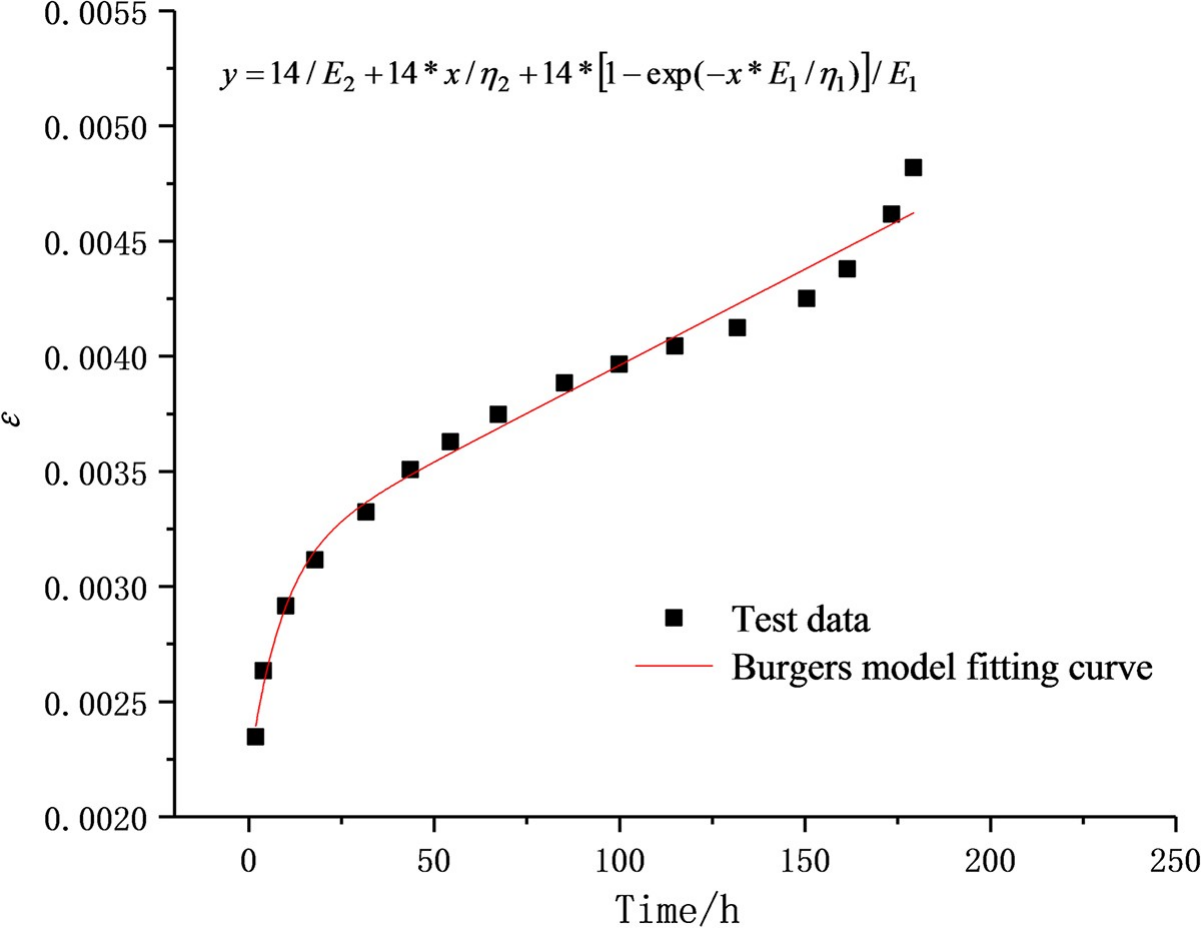
Fitting curve of Burgers model.

**Fig 13:**
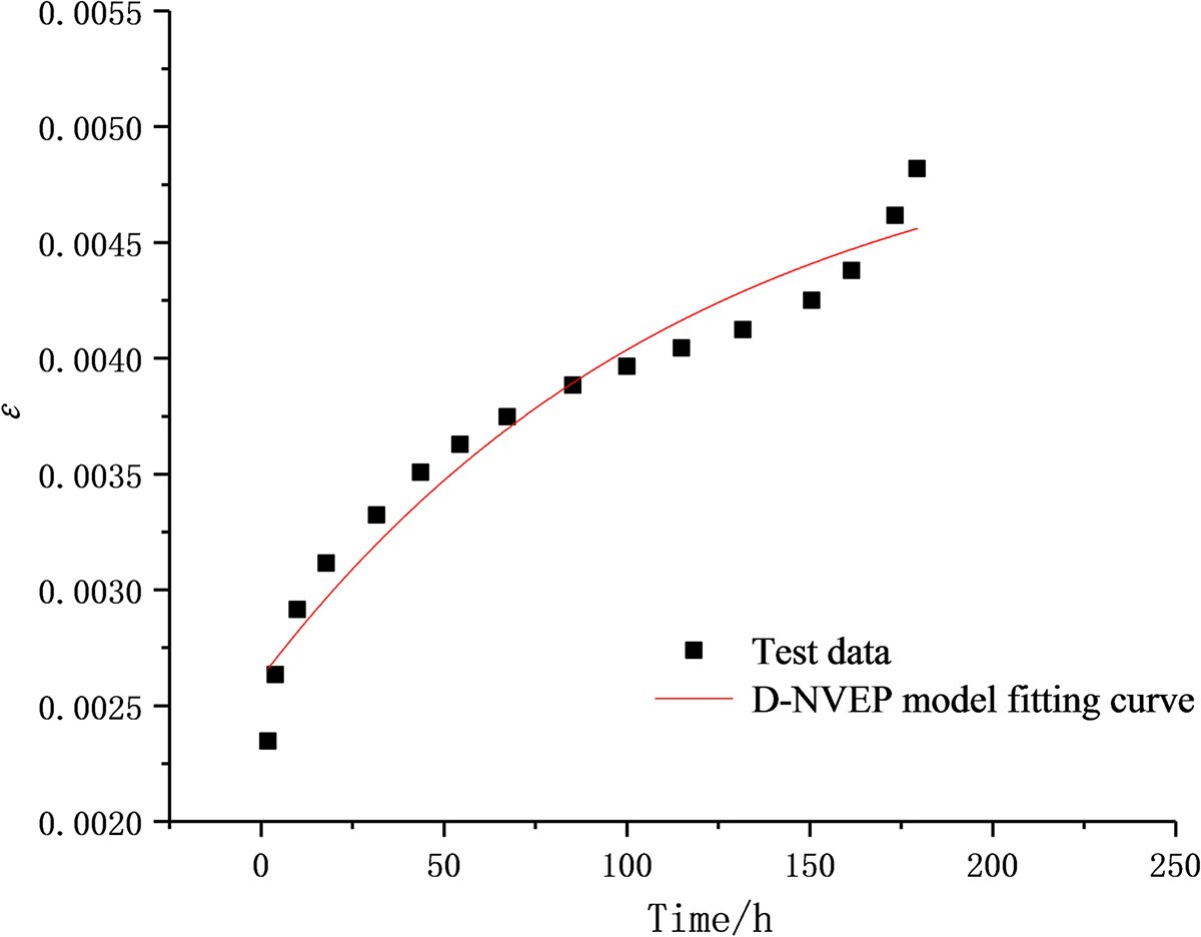
Fitting curve of D-NVEP model (without initial value).

The initial values of optimization parameters are the key to the success of the fitting process. The experimental data are therefore processed to separate the stable creep stage from the unsteady creep stage, which are respectively fitted with the Burgers creep equation and the D-NVP volume creep equation. As shown in Figs 9 and 10, after 41 iterations, the stable creep stage is successfully fitted with a correlation coefficient of 0.9915; after 22 iterations, the unsteady creep stage is fitted successfully with a correlation coefficient of 0.9889. The obtained model parameters shown in Tables 2 and 3 are used as the initial values of the optimization parameters for overall fitting. After 372 iterations of calculation and fitting, as shown in Figs 11 and 12, the Burgers model cannot describe the accelerated creep stage, whereas the D-NVEP creep model has good fitting effects in the attenuation, constant velocity and accelerated creep stages. The correlation coefficient is 0.9909, verifying the applicability of the D-NVEP creep model. When Eq (15) is directly used for the overall fitting, as shown in Fig 13, after the maximum number of iterations is reached (the default maximum number of iterations in the Origin software is 400), the system indicates that the fitting fails. The cause for the improved fitting results of the D-NVEP creep model may be interpreted as follows. The piecewise fitting model involves less parameters and the classical rheological model can better describe the attenuation and constant velocity creep curves. The identification parameters obtained by piecewise fitting can reflect the changing trend of the creep curve and are closer to the optimal solution of the iterative calculation. With the obtained identification parameters as the initial values, the successful overall fitting process can be easier made with the superposition method. The identified final model parameters are shown in Table 4.

**Table 2:** Identification table of model parameters in stable creep stage.

Stress /MPa	*E*_1_/MPa	*E*_2_/MPa	*η*_1_/MPa·d	*η*_2_/MPa·d	*R*^2^
14	1.483e4	6.276e3	1.529e5	1.767e6	0.9915

**Table 3:** Identification table of model parameters in unsteady creep stage.

Stress /MPa	*η*_3_/MPa·d	*h*	*g*	*R*^2^
14	6.479e4	-14.995	19.663	0.9889

**Table 4:** Identification table of CF-D-NVP model parameter.

Stress /MPa	*E*_1_/MPa	*E*_2_/MPa	*η*_1_/MPa·h	*η*_2_/MPa·h	*η*_3_/MPa·h	*h*	*g*	*R*^2^
14	841.511	3.245e6	6.315e4	3.921e7	2.123e5	-57.915	68.964	0.9909

**Fig 14:**
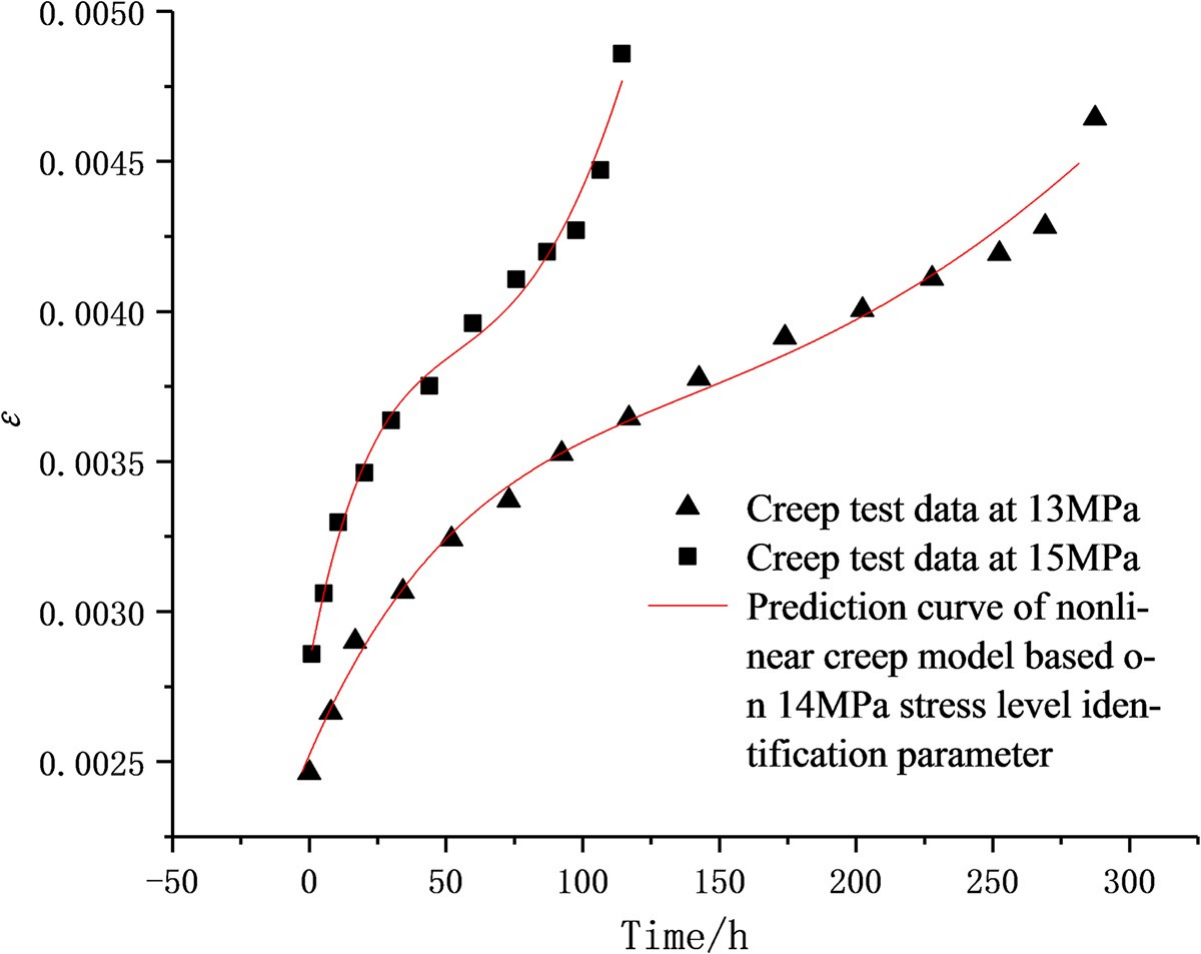
Comparison between the predicted curve and the test curve under stress levels of 14 MPa and 13 MPa.

The D-NVEP creep model is further verified as follows. Since the three groups of samples in the previous study [16] were taken from mudstone in the same area, it is considered that the lithology of the samples is approximately the same. According to the identification parameters of the D-NVEP creep model with a stress level of 14 MPa (Table 4), creep curves under the stress levels of 13 MPa and 15 MPa are predicted. The comparison between the predicted curve and the test curve is shown in Fig 14. Due to the difference of the initial damage such as original joints and cracks in the specimen and data fluctuation caused by collection time interval of test data, the prediction curve deviates from partial test values during the constant velocity creep stage and accelerated creep stage. However, the variation trend of the strain with creep time is basically the same, indicating that the D-NVEP creep model can be used to describe the creep characteristics under different stress levels.

## 6. Discussion

Due to different lithologies of different rock masses and the complexity of engineering geological environments, it is difficult to study rock creep with a unified model. It is necessary to propose a constitutive model describing the whole process of creep according to specific conditions. The research object of this paper is the mudstone interlayer, whose strength is lower than that of the upper and lower strata and the shear failure often occurs due to strength deterioration. Compared with the previous models based on the elastic modulus, viscosity coefficient or a separate damage factor of rock mass, the D-NVEP model proposed in this paper based on the damage characteristics of mudstone strength parameters is more suitable. Compared with the empirical constitutive model, the D-NVEP model has clear physical significance. According to the deformation characteristics of different creep stages, D-NVEP model is established by means of component combination with s simple principle and easy to be understood. Compared with establishing a nonlinear viscoplastic body from the perspective of viscous element to characterize the nonlinear characteristics of the accelerated creep stage, the introduction of the rock strength damage variable in the traditional plastic element can more intuitively reflect the creep mechanism of viscoplastic failures of mudstone interlayer caused by strength deterioration under the action of stress and time.

## 7. Conclusions

(1) When subjected to continuous stress higher than mudstone damage threshold, the strength of mudstone decays with time. With strength parameter as a function of stress and time, the damage evolution process during mudstone creep deformation is described quantitatively to reveal the non-linear viscoplastic characteristics of mudstone.

(2) Based on the damage degradation relationship of cohesive force and internal friction coefficient with stress and time in mudstone creep tests, a new nonlinear damage viscoplastic body (D-NVP body) is established. The D-NVEP creep model is then established to characterize the three-stage characteristics of the complete creep period including the accelerated creep stage, which cannot be described with traditional rheological models.

(3) The LM-nonlinear least squares method is used to perform the fitting analysis through piecewise fitting and overall fitting so as to avoid the problem of non-convergence of the fitting caused by improper setting of initial values of identification parameters.

(4) Creep test data of mudstone verified the applicability and feasibility of the D-NVEP creep model.
